# E-Skin Using Fringing Field Electrical Impedance Tomography with an Ionic Liquid Domain

**DOI:** 10.3390/s22135040

**Published:** 2022-07-04

**Authors:** Manuchehr Soleimani, Myron Friedrich

**Affiliations:** Engineering Tomography Laboratory (ETL), Department of Electronic and Electrical Engineering, University of Bath, Bath BA2 7AY, UK; mf586@bath.ac.uk

**Keywords:** electrical impedance tomography (EIT), robotic skin, pressure mapping imaging, fringing field imaging, touch sensing

## Abstract

Electrical impedance tomography (EIT) is a promising technique for large area tactile sensing for robotic skin. This study presents a novel EIT-based force and touch sensor that features a latex membrane acting as soft skin and an ionic liquid domain. The sensor works based on fringing field EIT where the touch or force leads to a deformation in the latex membrane causing detectable changes in EIT data. This article analyses the performance of this electronic skin in terms of its dynamical behaviour, position accuracy and quantitative force sensing. Investigation into the sensor’s performance showed it to be hypersensitive, in that it can reliably detect forces as small as 64 mN. Furthermore, multi-touch discrimination and annular force sensing is displayed. The hysteresis in force sensing is investigated showing a very negligible hysteresis. This is a direct result of the latex membrane and the ionic liquid-based domain design compared to more traditional fabric-based touch sensors due to the reduction in electromechanical coupling. A novel test is devised that displayed the dynamic performance of the sensor by showing its ability to record a 1 Hz frequency, which was applied to the membrane in a tapping fashion. Overall, the results show a considerable progress in ionic liquid EIT-based sensors. These findings place the EIT-based sensors that comprise a liquid domain, at the forefront of research into tactile robotic skin.

## 1. Introduction

Human robotic interactions (HRIs) are becoming ever more prevalent as robot functionality increases [1]. Tactile sensing plays a crucial role in HRI for two main reasons. Firstly, tactile sensing provides a robot with additional proprioceptive feedback to allow for safer interactions [2]; for example, to give a robot information as to whether large amounts of pressure are being applied to a body (or a human) as a direct result of the robot’s movement. Secondly, tactile sensing can provide additional information as to how a human is communicating with a robot [3]; for example, to recognise whether a particular scenario was a gentle touch or a grab. This allows for more complex HRIs, where emotional recognition is required. Electrical impedance tomography (EIT) is an imaging technique that involves calculating the conductivity distribution of a medium, based on voltage measurements from electrodes, placed at known locations on the medium’s boundary [4]. In recent years, EIT has been a focus of research into electronic skins for the field of robotics, as it offers continuous, large area sensing with minimal wiring [3]. The EIT-based pressure sensors feature a conductive domain that needs to be flexible and piezoresistive [3]. Originally these domains were made of conductive rubber [3], however, designs that comprise a room temperature ionic liquid-based domain are emerging with promising results [5,6]. The key feature of these new fluid-based EIT pressure sensors is their self-healing characteristics [5], which allows for a dynamic performance, unparallel by any other EIT-based sensor.

Electrical impedance tomography was invented in the early quarter of the 20th century, by Conrad Schlumberger, as a form of underground electrical prospecting [7]. Schlumberger inserted large electrodes into the ground and applied a direct current between certain electrodes, while measuring the voltages between other electrodes [7]. These voltage measurements were used to infer and map the conductivity distribution below the surface of the ground, with the purpose of finding and monitor oil wells [7]. The application of EIT spread to medical engineering in 1978 when Henderson and Webster published the first impedance image of human tissue [8]. Electrical impedance tomography is attractive in clinical applications because it is non-invasive and non-destructive [9]. The EIT-based pressure sensors were introduced to the field of robotics by Kato et al. [10] and Nagakubo et al. [11] in 2007. Their designs used sheets of rubber that were coated with a conductive material to create domains that experience local changes in conductivity due to deformation because of applied pressure [10,11]. The sensors offered tactile pressure sensing, in that they could detect multiple stimuli across the domain simultaneously, in a continuous manner [10,11]. As the designs were comprised of flexible materials, and EIT can be applied to any two-dimensional shape, the sensors presented an innovative technology for pressure sensitive robotic skin [3]. The alternative to such artificial skins, is to use an array of discrete sensors, however, the new EIT-based technology offers the following advantages: minimal wiring; flexible; scalable; continuous sensing across the domain; easy to manufacture; low power consumption and low cost [3,12]. The e-skin has become a very important area of development in the past few years [13,14,15,16,17,18], whether it is for robots that are like humans, or robots that needs to work in a safe environment with humans or other robots. This paper covers an important area of soft robotics by providing methods of performance evaluation. The advantage of the tomographic approach, where few sensors can provide large amount of information with the aid of tomographic regularization and prior assumptions, will allow this to be a very efficient distributed e-skin sensor.

Conductive fabrics and other types of piezoresistive sensors have been the focus of EIT-based pressure sensor research in recent years, as the rubber domains of the original sensors gave very low changes in conductivity in response to pressure and showed high hysteresis [3]. Additionally, the conductive fabrics offered a stretchability that the rubber domains did not; this also posed a problem however, as the conductive fabric domains would experience local changes in conductivity because of stretch that were indistinguishable from the changes due to pressure [3]. In 2015, an EIT-based pressure sensor comprised of a flexible insulating material (silicon) with an embedded network of microchannels was introduced by Chossat et al. [19]. The microchannels contained a room temperature ionic liquid which acted as the conductive domain [19]. The advantage of such a sensor over its predecessors was in the predictability and repeatability of the conductivity changes that result from pressure deformation because the changes were merely a result of changes in the microchannel geometry, rather than that of complex physical phenomena [19]. However, the use of a network structure discretised the sensing domain. In 2020, Zhao et al. prototyped an ionic liquid-based EIT pressure sensor that featured a continuous domain [5]. Numerous studies on challenging aspects of EIT pressure sensing are shown in [13,20,21,22,23] dealing with sensor design, sensor performance and image enhancement. A systematic comparison between fabric- and liquid-based skin could be an interesting future study.

This paper describes an investigation into EIT pressure sensors, specifically for their applications in the field of robotics, as artificial tactile skins. The investigation can be dissected into the following main aims: There are few recent publications on this type of liquid-based sensor, so creating a functional EIT pressure sensor that comprises a liquid domain will act as validation of the previous work reported in the literature. The desired unique selling point of the sensor design will be its hypersensitivity, a feature that is highly valuable for safe critical applications of the sensor as a robotic skin. An in-depth performance investigation is needed to assess the performance of the sensor. Comparison to existing liquid-based EIT pressure sensors is needed to assess its performance and assess the value of the design. The data acquisition system of the EIT provides 16 frames/sec that together with image reconstruction allows 3 frames of EIT data per second to be recovered. In the following section, we will show the detection of very small forces. The hypersensitivity of the sensor and better than 3 Hz response for both the skin sensor and the EIT device will make this sensor a viable e-skin for collaborative robotic environment.

The dynamic performance of a liquid-based EIT pressure sensor has not been explicitly shown in the literature. The results from a quantitative method of assessment would be of great value to this field of research. In Section 2, a brief description of the EIT hardware and the sensor design is given together with a description of EIT image reconstruction. Section 3 focuses on static experiments considering multiple touch detection, accuracy of recovered touch position and the use of the sensor in horizonal and vertical settings. Section 4 is dedicated to quantitating force analysis and a position compensated calibration method for force quantification. Section 5 entails an investigation into the dynamical performance of the sensor. If the sensor is considered for the HRI application, the dynamical performance is critical, for example social robotics will requires a low latency response for unwanted collisions and the temporal function of touch sensing. Conclusions are drawn in Section 6.

## 2. EIT Imaging System

**Figure 1:**
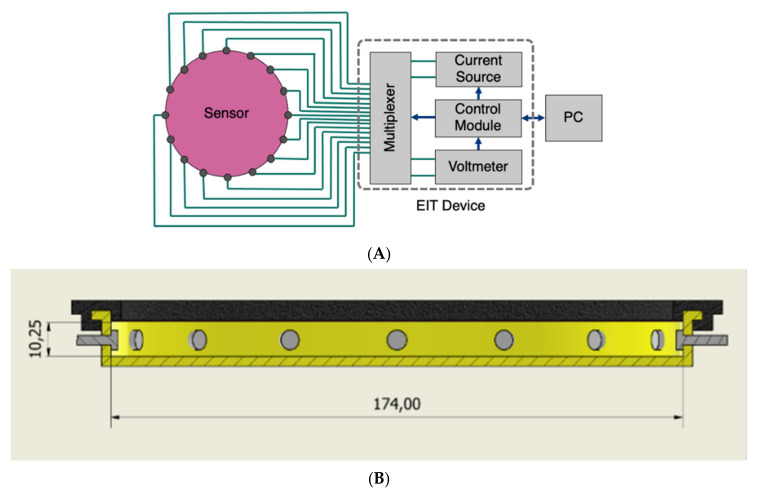
(**A**) block diagram of hardware system (**B**) CAD drawing of the EIT pressure sensor design, dimensions given in mm.

An EIT device that was created for monitoring wastewater flow in pipes [24,25,26] was used. The device injects a 50 kHz 6 mA current using an adjacent pair drive pattern for 16 electrodes. With 16 electrodes, 208 measured data points can be collected for each imaging frame. The device can produce 16 frames per second. Figure 1A shows a block diagram of the EIT system including current source, voltage measurement and switching multiplexers controlled with microcontroller through a computer. The signal-to-noise ratio (SNR) is useful to devalue the reliability and repeatability of the measured data. The SNR of the system was calculated over 100 frames of background data using

(1) $${\mathrm{SNR}}_{dB}=\frac{1}{N}\overset{N}{\underset{i=1}{\sum }}10\log\left(\frac{\frac{1}{K}\sum_{j=1}^{K}\left(V_{i,j}\right)}{\sqrt{\frac{1}{K}\sum_{j=1}^{K}\left({\left(V_{i,j}-\bar{V_{i}}\right)}^{2}\right)}}\right)$$

where N is the number of voltage measurements in one frame (208), K is the number of frames that the signal-to-noise ratio was calculated over (100 in this case) and V is the collection of voltage measurements acquired during data acquisition. An average SNR of 59.7 dB was obtained.

For EIT image reconstruction, a standard Tikhonov regularization algorithm, was used [25]. The forward problem in EIT is to find the boundary potentials with a given injected current, for a known (uniform) conductivity distribution [3]. The forward solution is obtained by solving

(2) $$\nabla \cdot \left(\sigma \nabla u\right)=0$$

where σ is the conductivity and u is the voltage, using the finite element method (FEM) with the complete electrode model, in which the continuous domain is discretised into a mesh of elements interconnected by nodes [3]. The inverse problem involves calculating the conductivity distribution of the domain for a given current injection pattern and known (measured) boundary potentials [4]. Dynamic imaging, or difference EIT, is a non-iterative method for calculating only the changes in conductivity across the domain, instead of the absolute values [9]. This is sufficient for artificial skin applications and is preferred (over static imaging) for its computational efficiency. The technique relies on a precalculated sensitivity matrix (or Jacobian) that is obtained using the forward solution [3]. A linear (approximation) Jacobian was calculated using EIDORS [12] and the image reconstruction was performed on MATLAB using the standard Tikhonov regularization algorithm [4]

(3) $$\delta \sigma ={\left(J^{T}J+\alpha^{2}R^{T}R\right)}^{-1}\;J^{T}\delta V$$

where α is the hyperparameter which controls the level of regularisation (α=0.00001 was used), R is the regularistation matrix (a Laplacian-type matrix was used) and J is the Jacobean representing the sensitivity matrix.

A CAD drawing of the final sensor design is given in Figure 1B with a 3D model depicted. The sensor comprises five components: the base, designed from the phantom tank; 16 transmitting/sensing electrodes, that are M3 bolts (with nuts and washers on the inside), spaced equally around the domain; an ionic liquid, that is tap water with added salt to bring the impedance between adjacent electrodes into the range required by the EIT device; a membrane, made of 0.4 mm thick latex and a rim clip to fasten the latex in place. The base and rim clip were 3D printed in ABS and TPU plastics, respectively. As before, the ABS tank had to be lacquered to make it waterproof. The ABS is rigid whereas the TPU is flexible, this allows for the rim clip to pop onto the flange of the base, creating a fit that is tight enough to seal the liquid in the sensor. The liquid fills the entire space enclosed by the latex and the base; this, combined with the fact that the latex is highly elastic (0.4 mm thick latex), gives the active region of the sensor self-healing characteristics, like that of real human skin, as required from the design.

The sensor was designed to be of minimum thickness, as this is a design requirement of robotic skin. The liquid-based sensor has a depth of 10.25 mm, this is simply the minimum depth that will allow for the M3 bolts (with washers) to fit into the domain. We observed that placing the electrodes nearer to the upper surface of the domain gave a higher sensitivity and use of small electrodes allows a better capture of fringing fields while requiring small height of liquid for the skin. Although using smaller electrodes, placed just in the upper region of the domain is likely to give a higher sensitivity, it is unknown if reducing the electrode size would have an impact on the signal-to-noise ratio of the device. Therefore, the previously tested bolts were selected.

## 3. Static Imaging

**Figure 2:**
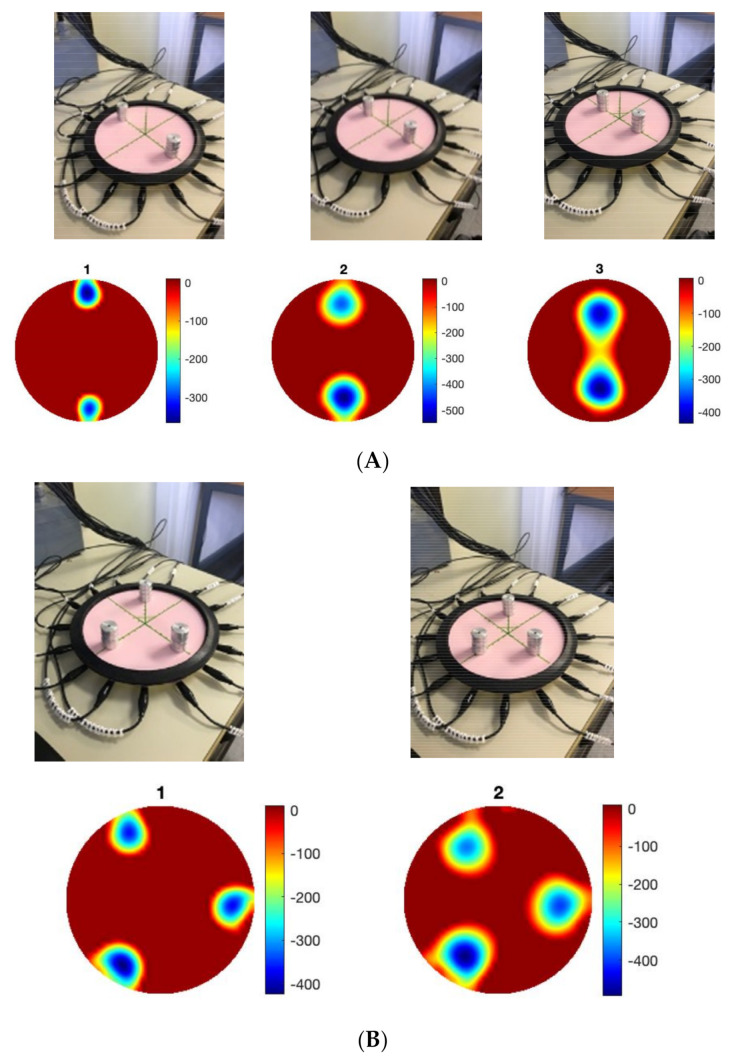

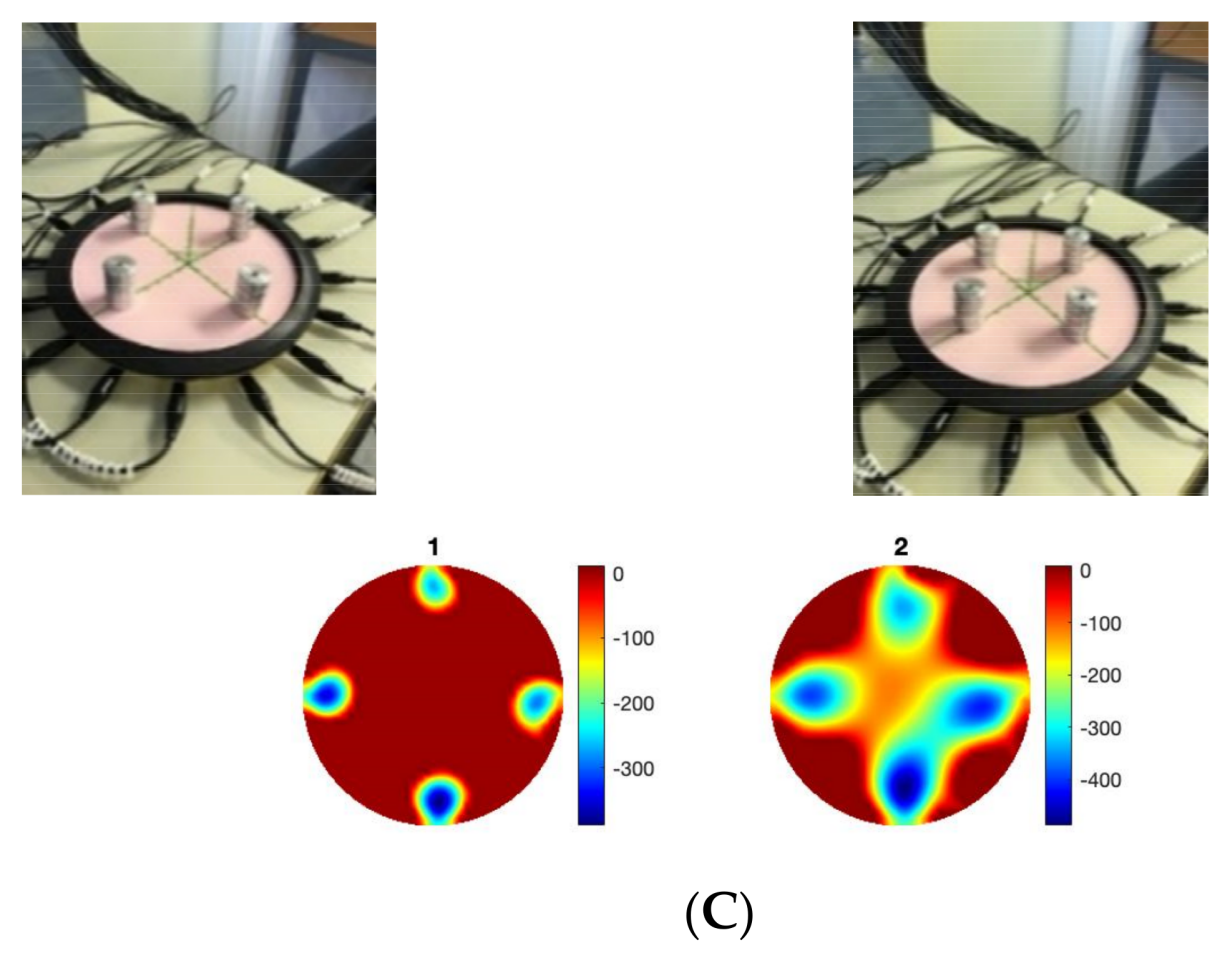
Sensing multi touch points, (**A**) two points, (**B**) three points, (**C**) four points.

The results discussed in this section form the basic performance investigation for the EIT pressure sensor. Two-point, three-point and four-point discrimination tests were performed on the sensor as shown in Figure 2. The pressure points were placed at equal radiuses so that the scale of each inclusion in the images would be of a similar magnitude. Each of the pressure points were created by placing a stack of four 6.5 g weights on the sensor, giving each point an equivalent force of 0.255 N. The weights had a diameter of 20 mm, so the equivalent pressure at each point was 0.812 kPa.

**Figure 3:**
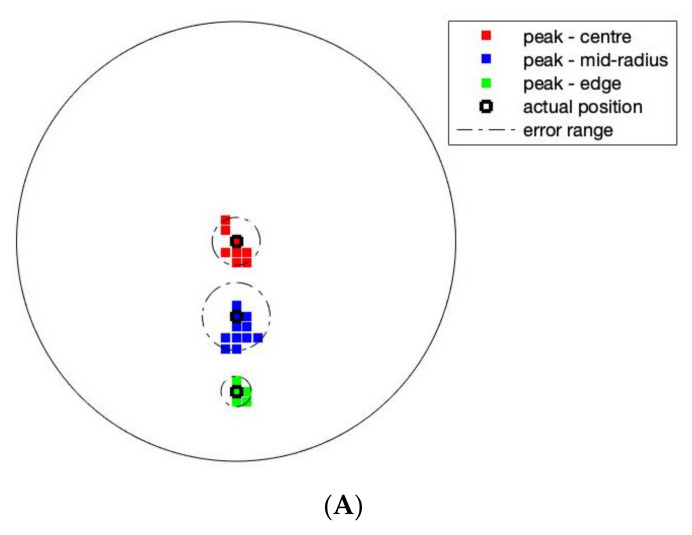

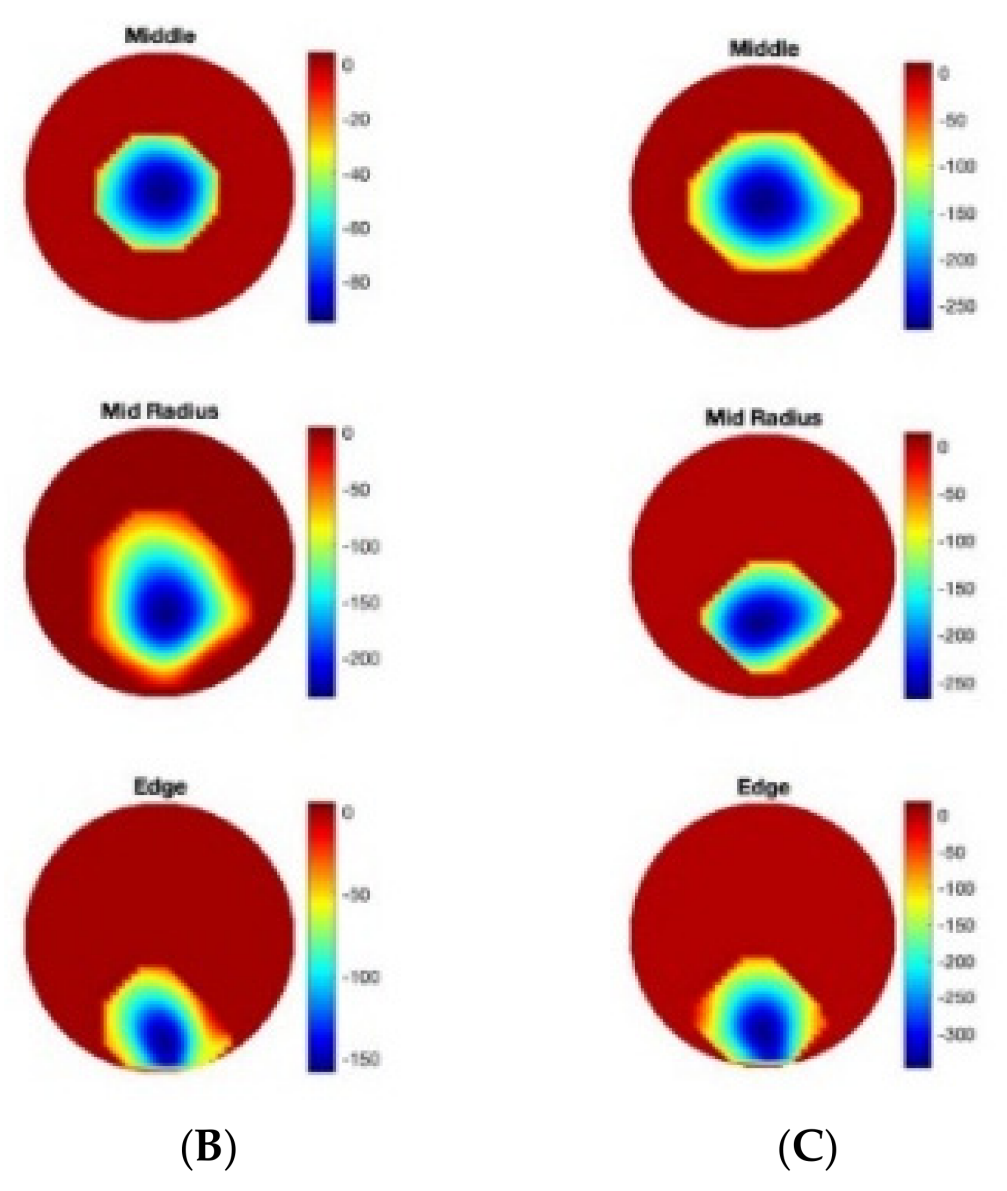
(**A**) Target position accuracy and reconstruction of (**B**) reconstruction of force if 64 mN, (**C**) force of 128 mN.

The sensor was able to discriminate up to four points without any additional image processing. For tactile robotic skin, it is preferable to detect multiple inclusions; for an EIT-based sensor, four-point touch is acceptable [22]. Over a circular domain of only 174 mm diameter, four-point discrimination is sufficient for safety applications of robotic skin but may be insufficient for HRIs that involve emotional recognition. This is an important area of soft robotics [27], which was investigated in the context of the EIT skin in [28]. The location of touch, the intensity of touch and its dynamics are all important for the social and interactive robotics future. Here, we investigated the position accuracy. The incremental weight test data contains a total of 108 images (3 locations × 4 repeats × 9 different weights) at three radial locations (0, 30 mm and 60 mm from the central position). The 96 images of 2–9 weights, giving a force ranging from 0.128 N to 0.58 N, were used to investigate the positional error of the sensor. Due to the smoothness of the images, the peak value of the image is taken to represent the centre of an inclusion in the image. Figure 3A shows the position detection for pressure point in various places. A maximum error of 6 mm can be observed, this error could be higher for smaller forces. Figure 3B,C shows a small detectable force of 64 mN and 128 mN can be seen in various locations; the smallest force is 64 mN and a pressure of 0.203 kPa.

**Figure 4:**
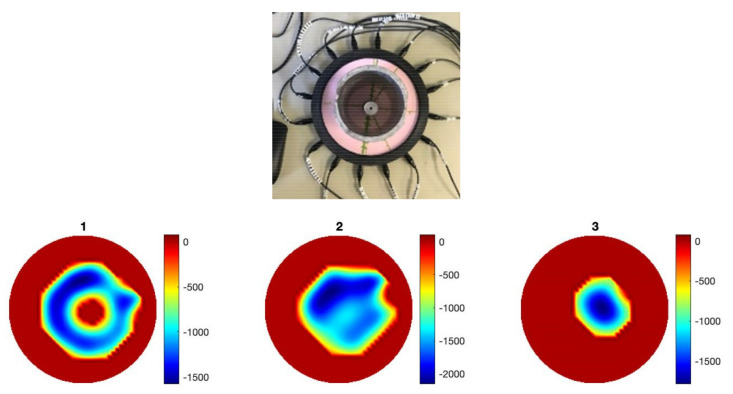
An annular force sensing, detecting the ring and the force within the ring. Image 1 is a reconstruction of ring shape, image 2 a reconstruction of ring and an internal touch point, and image 3 reconstruction of touch point inside ring.

**Figure 5:**
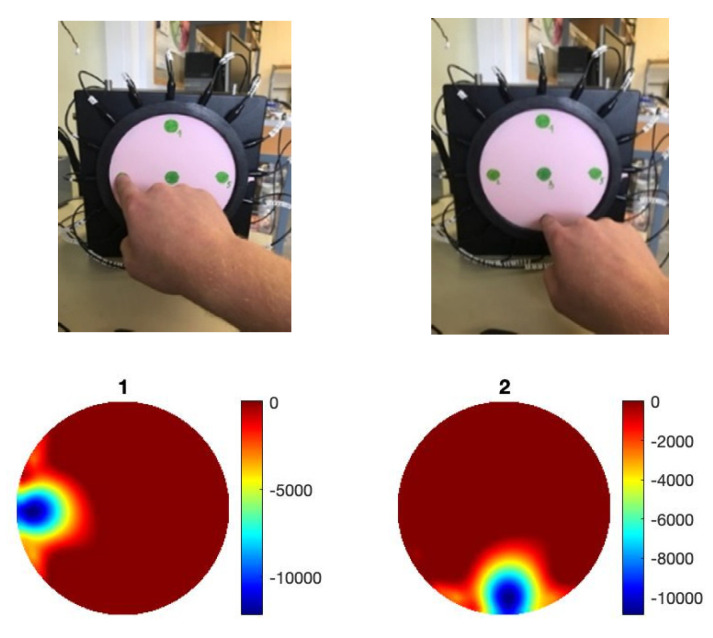
Skin sensor in vertical stand, points of touch shown in south, north, west and east. A point of touch on the west side (left) and a point of touch on the south side (right).

**Figure 6:**
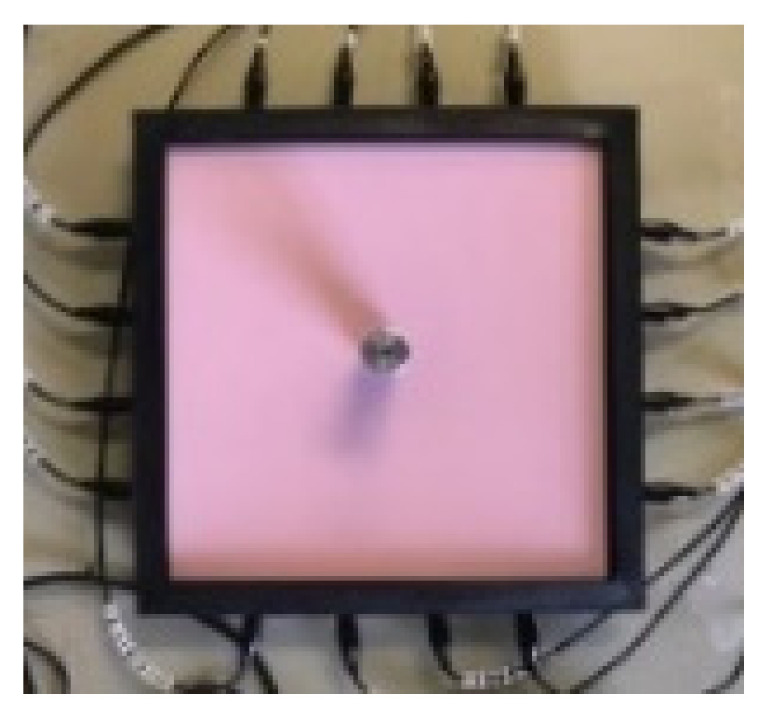

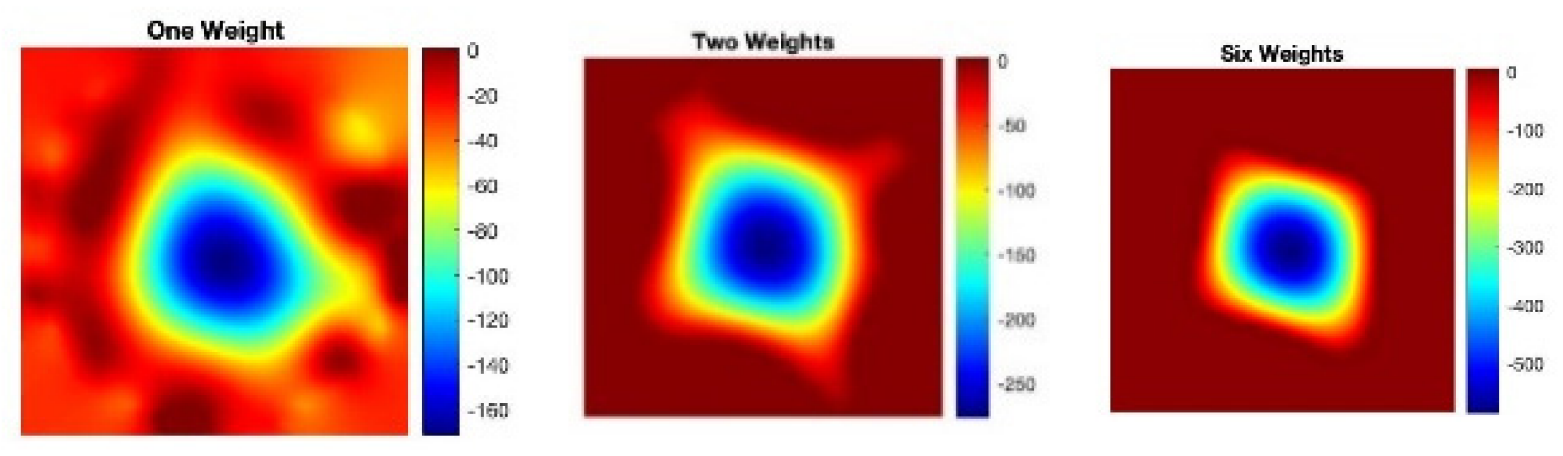
Square shape sensor, EIT image from left to right for 6.5 g, 13 g and 39 g weight.

Imaging through the fringing field allows detection of annular shape force without fully blocking the EIT signals. As shown in Figure 4, an inclusion within an annular force can be detected. Figure 4 shows images when the reference data is relaxed skin and a circular pressure is applied, when a circular and annular object with no pressure is used as a reference, and when a ring was chosen as a reference so an internal object can be detected. The ability of the sensor to detect the annular region of pressure may also have significant value in robotic skin applications involving emotional recognition. For example, a palm of a hand would make a similar region of pressure. The fact that this detection was only possible because of the fringing field of the device suggests that liquid domains with similar membrane designs are at the forefront when it comes to building more complex images of pressure. Although no conclusions could be made as to the effect of the domain’s shape on the performance of the sensor, the square sensor investigation did yield positive result. The square shaped sensor proved to be as sensitive as circular array despite the increase in the sensing domain’s size; this suggests that the design can be scaled to some extent. Since the sensor is filled entirely with the ionic liquid, it is possible to use it in a vertical orientation as shown in Figure 5. The liquid-based EIT sensor can be developed in various geometries. Figure 6 shows an example of a square shaped sensor where the imaging of various forces 6.5 g, 13 g and 39 g are shown. The square sensor includes 16 electrodes and has a side length of 212 mm. It is worth noticing that in soft robotics applications of the EIT, many different types of sensor shapes may be used. This paper shows circular and square shaped sensors; theoretically, there is no challenge if those sensors are designed in different shapes and forms. The liquid-based EIT sensor would work more robustly if all electrodes were in touch with the ionic liquid and if there was no air gap in any location. The industrial EIT can work with half-filled pipes (i.e., some electrodes are not in touch with the conductive domain). In the context of e-skin, EIT liquid vacation can lead to some areas being not detectable. So, to avoid liquid vacation, the sensor can be designed in various geometries.

## 4. Quantitative Force Imaging

**Figure 7:**
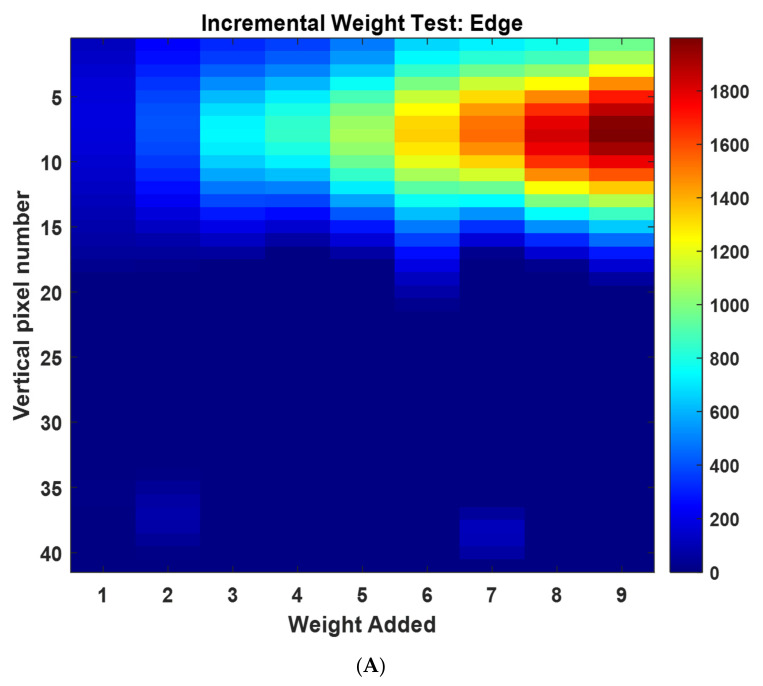

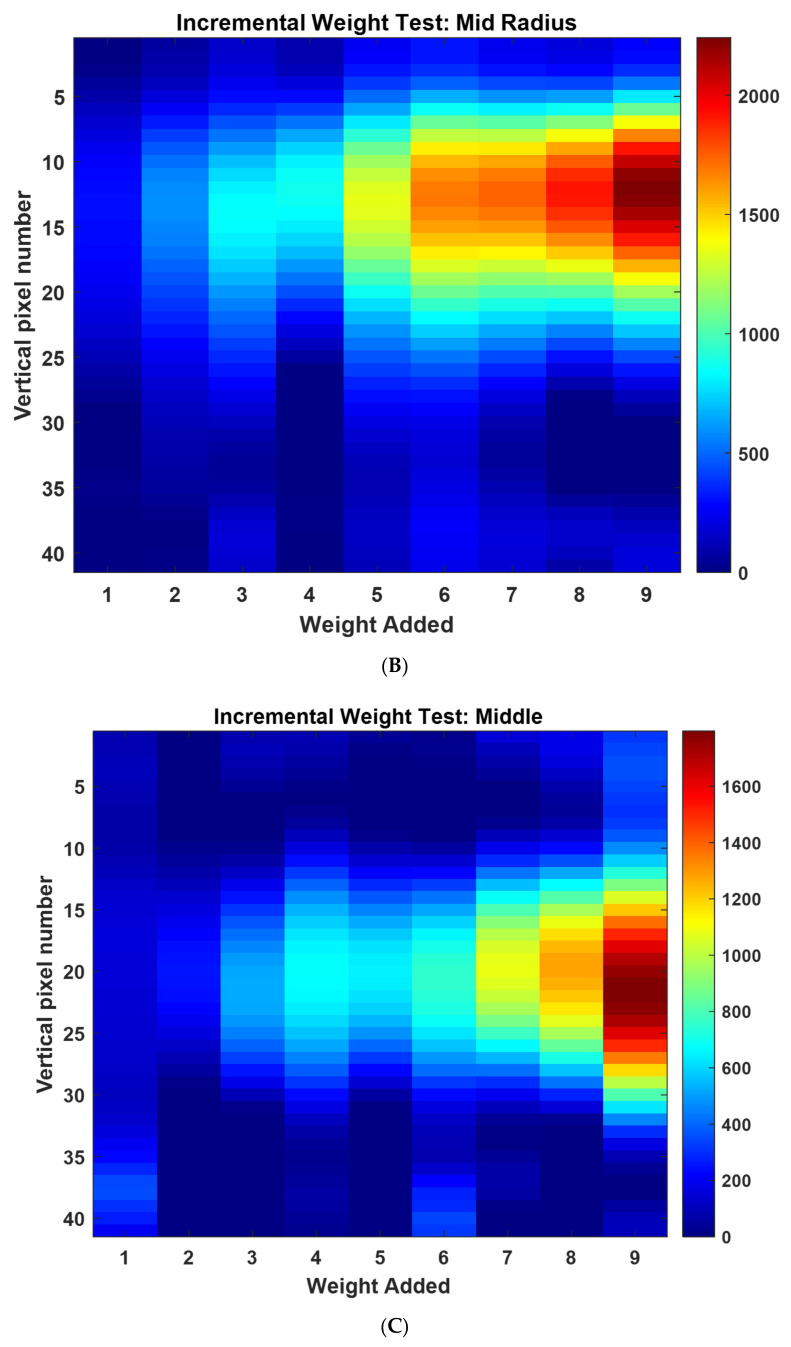
Sliced images with normalised colour-bar showing the vertical central pixel, a 40 × 40 imaging grid is used, so the radius is equal to 20 pixels (**A**) edge centred around 15 pixels from centre, (**B**) mid radius 10 pixels from centre, (**C**) centre.

First, we investigated the quantitative force analysis. The 6.5 g weights were stacked upon one another to create a single pressure point of constant area. The single inclusion was incremented from 1 to 9 weights creating mass from 6.5 g to 58.5 g. The equivalent force applied (by gravity) was incremented from 0.064 to 0.58 N. Due to the inherent location-dependant sensitivity, the experiment was performed at three locations of different radiuses, as shown in Figure 7. Images (40 × 40) were combined from nine experiments leading to volume data with the size of 40 × 40 × 9. Figure 7 shows a slice through this volume data through slice 20 in the y direction revealing the increase in image scale in the position of force with increasing weight.

**Figure 8:**
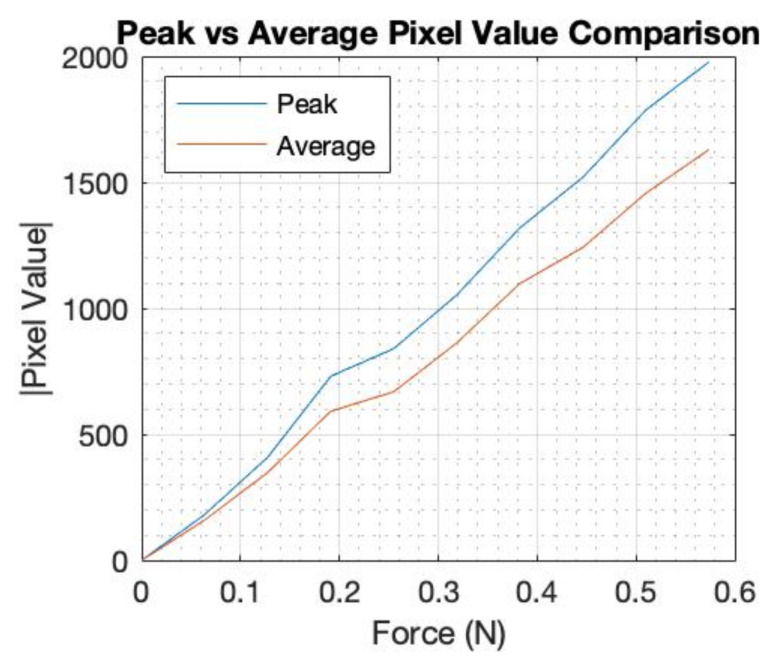
Force vs. peak or average image value.

Figure 8 shows a comparison of the peak and average pixel values for the incremental weight test at the edge location, where the average value was calculated over nine square pixels that surround the peak. This plot is included to show that the peak value is as robust as an average value calculated over a portion of the area of the inclusion. This is expected by the nature of Tikhonov regularization image reconstruction algorithm. The experiment was repeated twice, giving a total of three sets of data, where each image was an average of 10 images.

**Figure 9:**
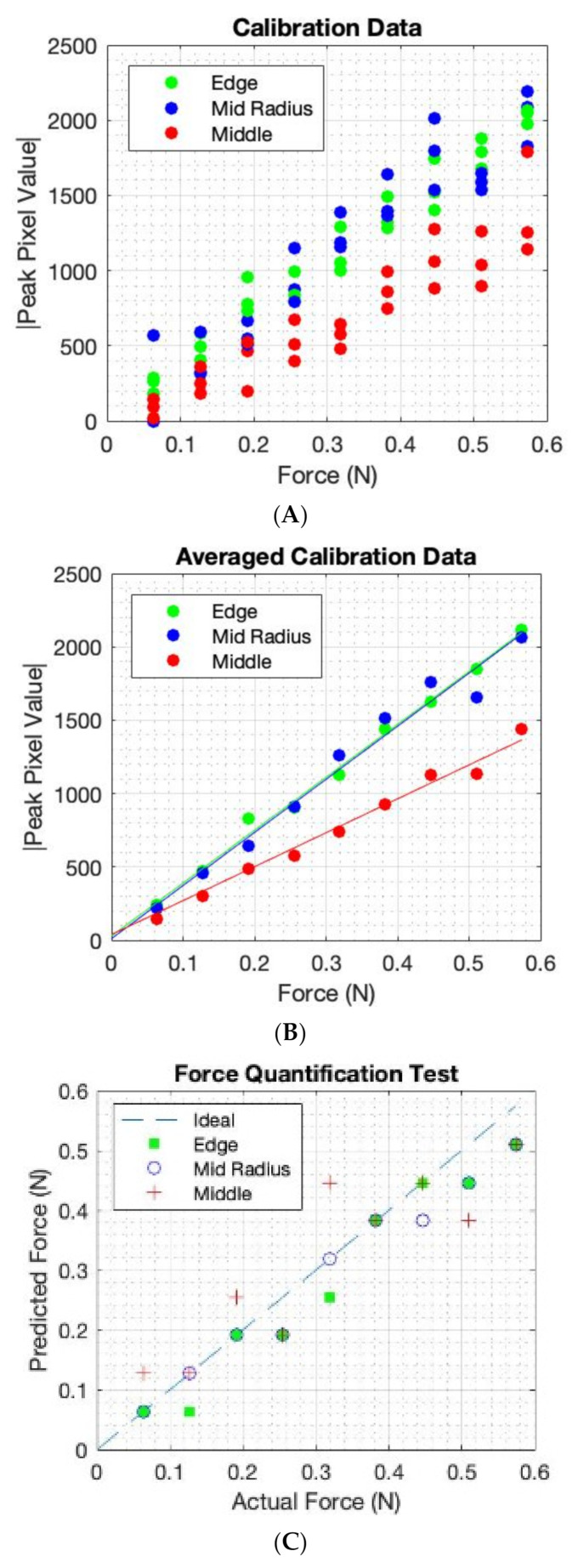
Calibration data for each position and an eventual linear calibration function (**A**) three points for each force (**B**) Average of three points, (**C**) location compensated calibration.

Figure 9 also shows the average of each of the three data sets, and the calibration lines that were generated for three positions. The mid-radius location gave a similar sensitivity to the edge; this is because, although the electrical sensitivity is lower at the mid-radius, the mechanical sensitivity is higher due to more freedom of deformation for the elastic latex. The EIT system in this design is sensitive to low forces. This was achieved by a fringing field EIT detection with small electrodes and a shallow phantom. Small deformation on soft latex skin produces a measurable change in EIT data. Small perturbation in EIT data, if it can be measured, leads to a close to linear EIT image reconstruction mode. This means that a linear force calibration is possible. By averaging three sets of data as shown in Figure 9B, we can establish a linear calibration function. In Figure 9C, by combining a location detection via the EIT image and the calibration setting in Figure 9B, we can produce a single calibration form as shown in Figure 9C. A data driven approach to force quantification is shown in [14]. Figure 9 shows an approach that combines the position of touch with the calibration leading to a single calibrating plot. This requires first an EIT image, and as shown in Figure 3, the position of touch can be detected and eventually leading to a single calibration graph. The force calibration can be done in many ways, for example, directly from the EIT measured data if a convolution neural network is used. It is also worth noticing that it is hard to produce quantities of EIT information in other application areas such as medical EIT and industrial EIT. Therefore, the fact that the soft skin EIT is showing an ability to quantify forces is a promising feature. For e-skin EIT, the image is still desired, so the proposed approach in this paper could be valuable.

**Figure 10:**
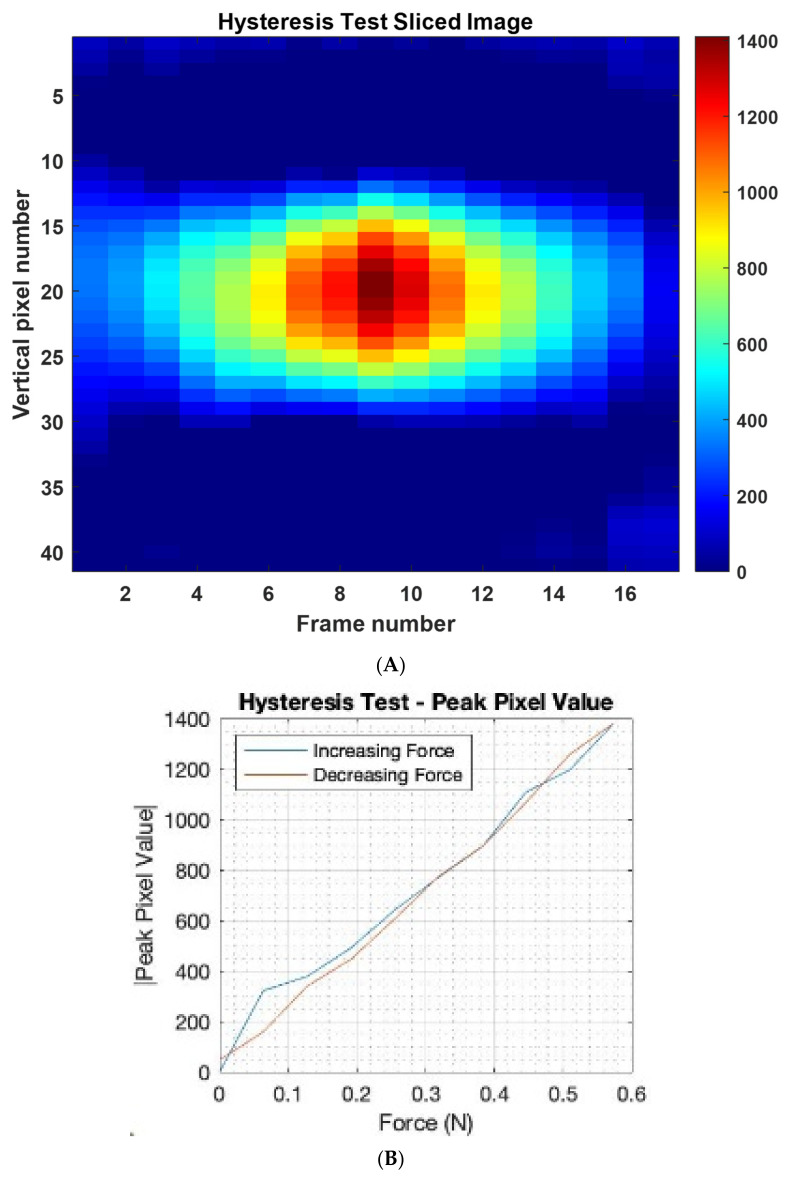
Adding and then removing weights in a systematic fashion for the hysteresis analysis, (**A**) image through slices with normalised colour-bar, the weight is systematically added and removed, added from frame 1-8, maximum at frame 9 and systematically removed from 10-18 (**B**) the hysteresis plot.

To investigate the hysteresis of the sensor, a single pressure point was created in the centre of the sensor using the 6.5 g weights from the previous experiments. We gradually increased the number of 6.5 g weights from 1 to 9 and then from 9 back to 1. Figure 10A shows the slice through the image and quantitative plot for force vs. the image peak value. The *x*-axis indicates increase and then decrease of weight of touching force. It should be possible to quantify the level of hysteresis by for example the area between the blue and red lines in Figure 10B. A lower the area between the ascending and descending curves would mean a thin hysteresis graph and a larger area indicates a thick hysteresis graph leading to more error, which can be a function of both the EIT measuring system and the performance of the e-skin sensor. As observed, the hysteresis shown in the imaging results are not high.

## 5. Dynamical Imaging 

**Figure 11:**
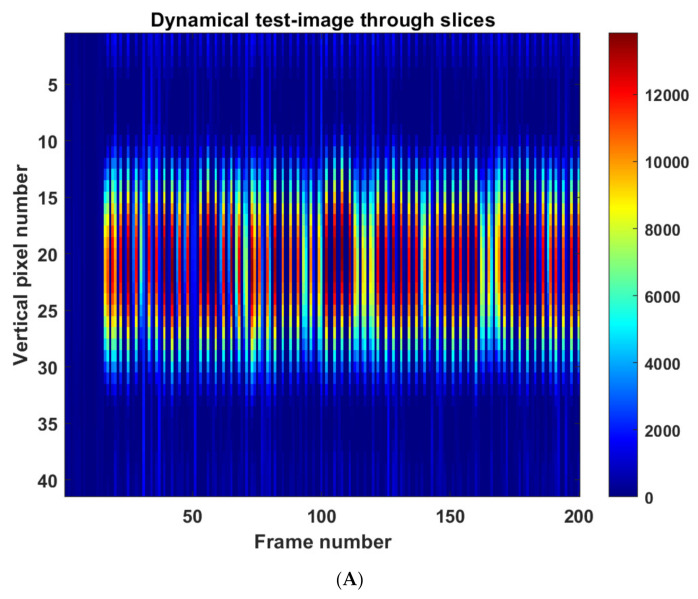

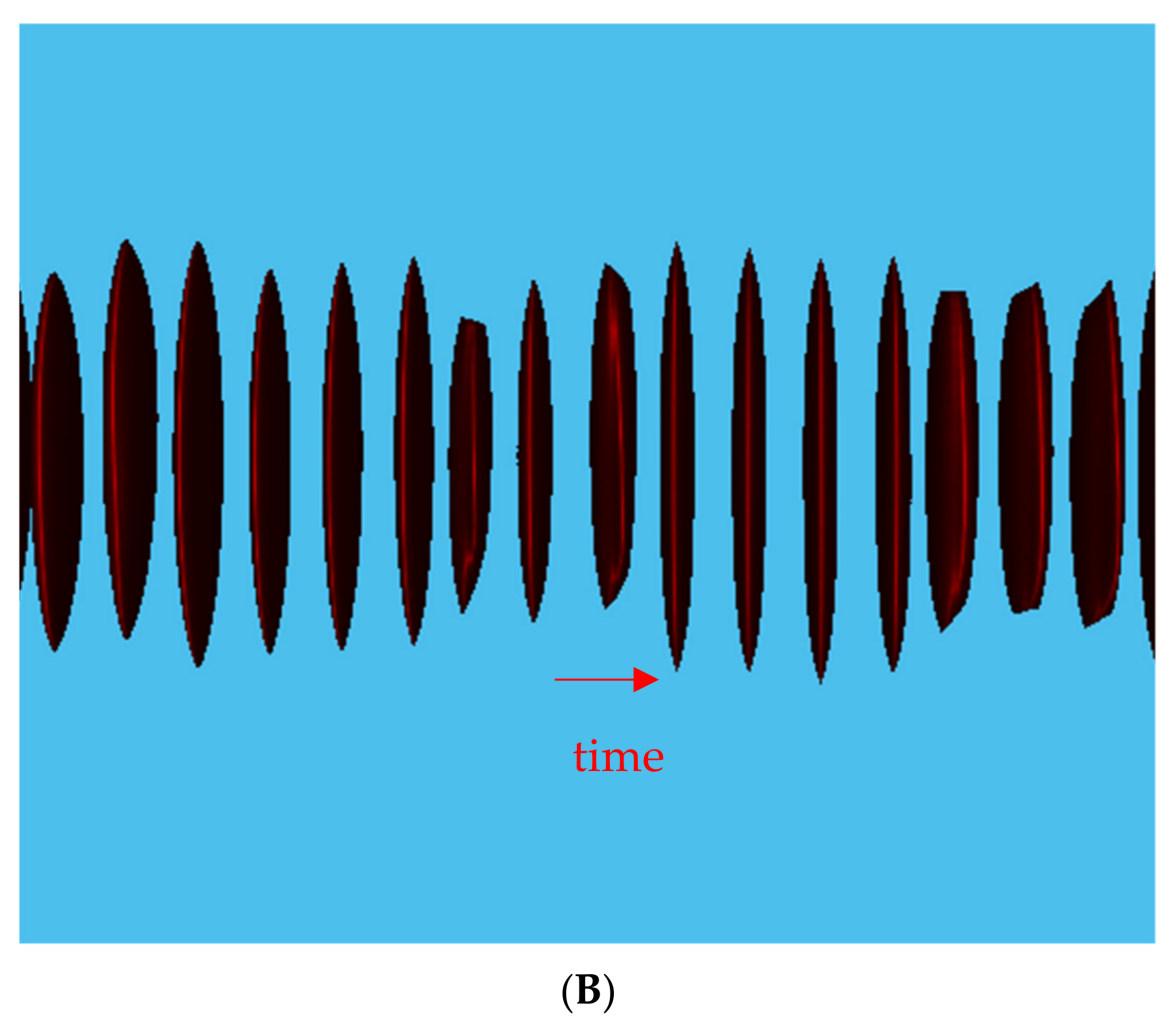
Dynamical touch with a frequency of 1 Hz, (**A**) slice through the images with normalised colour-bar, (**B**) volumetric representation of the pressure applied.

**Figure 12:**
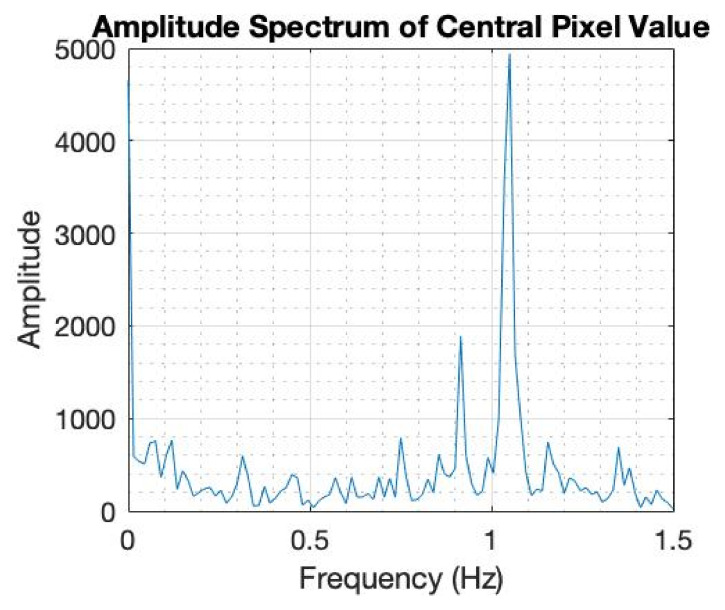
Inverse Fourier transfer to re-create the 1 Hz frequency of tapping.

To investigate the dynamic performance of the sensor, a test involving a regular frequency of touch was devised. In this case, no averaging of the data was performed during the test. A single pressure point was created in the central region of the sensing domain for alternating pressure application. An oscillatory motion was generated by removing and reapplying the pressure point in time with the ‘tick’ of a digital metronome. The metronome ‘tick’ had a frequency of 2 Hz; this was necessary so the participant applying the pressure point had a guide as to when to apply (1 Hz) and remove (1 Hz) the pressure point. If the pressure over the central region of the sensor were to be plotted against time, the waveform would be trapezoidal because of this oscillatory motion. The slice through the tapping image is shown in Figure 11; image slices are shown in Figure 11A. Figure 11B shows the temporal axes of the 3D rendition with the 2D images shown together with time in 3D. The central pixel value was extracted from the above image, and a fast Fourier transform was implemented to analyse the frequency information contained in the signal of the central pixel value, as shown in Figure 12. The frequency spectrum shows a clear peak at 1 Hz, validating that the oscillatory motion has been accurately captured. The fact that the frequency information was captured in this test, displays the rapid self-recovering characteristics achieved by the fluid and latex membrane. The dynamic performance of the sensor, displayed by its ability to record the 1 Hz frequency information in real-time, allows for complex spatial-temporal information to be captured. This could prove to be incredibly valuable in HRIs involving emotional recognition. This experiment was done by a human listening to sound notes of 2 Hz; for future study, a mechanism can be used to apply touch with a given temporal requirement.

## 6. Conclusions & Remarks

An in-depth analysis of an ionic liquid-based EIT-based tactile skin is presented. Many aspects of the skin sensor, including force quantification, the sensor’s dynamical response, the hysteresis and single and multiple touch detection were examined. The dynamic performance of the sensor was successfully shown using a novel test that involved capturing frequency information in real-time using the sensor. A 1 Hz signal was applied to the latex membrane of the sensor and successfully recorded and recovered. The sensor’s dynamic performance allows for complex spatial-temporal information to be captured, which will be critical for HRIs involving emotional recognition. This allows applications such as social robotics through sensing interactions via tactile skin. In addition to this, the hysteresis of the sensor was considered and shown to be very small. This is due to electromechanical decoupling between soft skin and the liquid, unlike the traditional fabric-based EIT skins: Due to the fringing field response, the sensor shows sensitivity to forces as small as 64 mN. The sensor’s hypersensitivity could be of great value in critical robotic interactive applications, where the smallest measurable force could be used to trigger a shutdown response if deemed to be a collision concerns. The ability to detect annular regions of pressure, puts the liquid-based EIT sensor design at the forefront when it comes to developing tactile robotic skin that can image complex shapes, such as a human handprint. When the ionic liquid completely fills the sensing volume, the sensor can work in horizonal or vertical positions adding to the versatility of the proposed skin. Future investigation could focus on different shaped sensors, the application with an autonomous system using sensing as part of the control, and a comparative study with fabric-based EIT sensors.

## Data Availability

Data could be available upon request.
